# Azithromycin targets the CD27 pathway to modulate CD27hi T-lymphocyte expansion and type-1 effector phenotype

**DOI:** 10.3389/fimmu.2024.1447625

**Published:** 2024-08-15

**Authors:** Abdul Wahid Ansari, Manju Nidagodu Jayakumar, Fareed Ahmad, Thenmozhi Venkatachalam, Laila Salameh, Hema Unnikannan, Thesni Raheed, Abdul Khader Mohammed, Bassam Mahboub, Basel K. Al-Ramadi, Qutayba Hamid, Martin Steinhoff, Rifat Hamoudi

**Affiliations:** ^1^ Research Institute for Medical and Health Sciences, University of Sharjah, Sharjah, United Arab Emirates; ^2^ Dermatology Institute, Interim Translational Research Institute, Academic Health System, Hamad Medical Corporation, Doha, Qatar; ^3^ Department of Pulmonary Medicine, Rashid Hospital, Dubai, United Arab Emirates; ^4^ Department of Medical Microbiology and Immunology, College of Medicine and Health Sciences, United Arab Emirates University, Al Ain, United Arab Emirates; ^5^ Zayed Center for Health Sciences, United Arab Emirates University (UAEU), Al Ain, United Arab Emirates; ^6^ ASPIRE Precision Medicine Research Institute Abu Dhabi, United Arab Emirates University, Al Ain, United Arab Emirates; ^7^ Department of Clinical Sciences, College of Medicine, University of Sharjah, Sharjah, United Arab Emirates; ^8^ Meakins-Christie Laboratories, Faculty of Medicine, McGill University, Montreal, QC, Canada; ^9^ Department of Dermatology and Venereology, Hamad Medical Corporation, Doha, Qatar; ^10^ Weill Cornell Medicine-Qatar, Doha, Qatar; ^11^ College of Medicine, Qatar University, Doha, Qatar; ^12^ College of Health and Life Sciences, Hamad Bin Khalifa University, Doha, Qatar; ^13^ Division of Surgery and Interventional Science, University College London, London, United Kingdom; ^14^ Biomedically Informed Artificial Intelligence Laboratory (BIMAI-Lab), University of Sharjah, Sharjah, United Arab Emirates; ^15^ ASPIRE Precision Medicine Research Institute Abu Dhabi, University of Sharjah, Sharjah, United Arab Emirates

**Keywords:** azithromycin, CD27 subset, T-lymphocytes, inflammation, mTOR, type-1 immunity, CXCR3

## Abstract

Macrolide antibiotic azithromycin is widely used in clinical practice to treat respiratory tract infections and inflammatory diseases. However, its mechanism of action is not fully understood. Given the involvement of the CD27 pathway in the pathophysiology of various T-lymphocyte-mediated inflammatory, autoimmune, and lymphoproliferative diseases, we examined the impact of AZM on CD27 regulation and potential consequences on CD4+ and CD8+ T-cell phenotypes. Using cellular immunology approaches on healthy donors’ peripheral blood mononuclear cells, we demonstrate AZM-mediated downregulation of surface CD27 expression as well as its extracellular release as soluble CD27. Notably, AZM-exposed CD27high (hi) cells were defective in their ability to expand compared to CD27intermediate (Int) and CD27low (lo) subsets. The defective CD27hi subset expansion was found to be associated with impaired cell proliferation and cell division. At the molecular level, the CD27hi subset exhibited lower mTOR activity than other subsets. Functionally, AZM treatment resulted in marked depletion of helper CD4+ (Th1) and cytotoxic CD8+ T-lymphocyte (Tc1)-associated CXCR3+CD27hi effector cells and inhibition of inflammatory cytokine IFN-γ production. These findings provide mechanistic insights on immunomodulatory features of AZM on T-lymphocyte by altering the CD27 pathway. From a clinical perspective, this study also sheds light on potential clinical benefits observed in patients on prophylactic AZM regimens against various respiratory diseases and opens avenues for future adjunct therapy against Th1- and Tc1-dominated inflammatory and autoimmune diseases.

## 1 Introduction

Azithromycin (AZM) is one of the highly prescribed macrolide antibiotics to treat airway diseases. In addition to their antimicrobial properties, AZM is also known to exert anti-inflammatory effects. For example, low-dose prophylactic treatment with AZM has shown clinical benefits by reducing exacerbations in chronic obstructive pulmonary disease (COPD) (1), asthma (2), idiopathic pulmonary fibrosis (IPF) (3), and non-cystic fibrosis bronchiectasis (4). These anti-inflammatory effects of AZM are mainly attributed to their ability to effectively inhibit pro-inflammatory cytokine production, reduction in lung neutrophil influx, and regulation of macrophage function (5–9). One of the major advantages of AZM is their retention within tissues for a longer period (10, 11). Some of the mechanisms of AZM-mediated anti-inflammatory responses include downregulation of pro-inflammatory cytokines IL-1β, TNF-α, and CXCL8 by inhibiting NF-kB signaling (12–14), suppression of the inflammasome (15), and polarization of macrophages to a regulatory M2 phenotype (16). In contrast to myeloid lineages, AZM is reported to inhibit T-cell activation by suppressing mammalian target of rapamycin (mTOR) signaling (17–19). Moreover, *in vitro* study has also demonstrated the suppression of TCR-activated helper CD4+ T cells (Th0) that may potentially affect subsequent differentiation of Th1 and Th2 cell lineages upon AZM exposure (20).

CD27 (TNFRSF7) belongs to the tumor necrosis factor (TNF) receptor superfamily expressed mainly by CD4+ and CD8+ T cells, B cells, and NK cells (21, 22). It is abundantly expressed on naïve and central memory and lose their expression on the effector memory T-cell subset following activation (23). CD27 is considered as a reliable memory T-lymphocyte and differentiation marker (24, 25). CD70 is the only known receptor of CD27, expressed mainly on activated B cells and to some extent on antigen-presenting cells (APCs) (26). Activation of CD27 is known to promote CD4+ and CD8+ T-cell expansion, survival, and memory cell generation (25, 27–29) via signaling involving TRAF2 and TRAF5-mediated JNK, NF-kB activation (30, 31), and PIM1 kinases (32). Upon stimulation, CD27 promotes differentiation of CD4+ T cells into helper type-1 (Th1) effectors (33) and CD8+ T cells into cytotoxic T-lymphocytes (Tc1) (34) by inducing type-1 transcription factor T-bet to transcribe type-1 signature cytokine interferon-gamma (IFN-γ) and chemokine receptor CXCR3 (35, 36).

Apart from their surface expression, the soluble form of CD27 (sCD27) is extracellularly released upon TCR activation via proteolytic cleavage of membrane-bound CD27 by metalloproteinase (37), which is evident in the clinical specimens of patients with autoimmune inflammatory diseases such as rheumatoid arthritis (RA) (38)and systemic lupus erythematosus (SLE) (39). Beside driving RA and SLE pathogenesis, Th1- and Tc1-secreted IFN-γ play a key role in inflammatory bowel diseases (IBD) (40, 41). In addition to cell-mediated immunity, CD27 effectively contributes to humoral immunity as well by interacting with its ligand CD70 expressed on activated B cells. Studies have demonstrated the involvement of CD27–CD70 interaction in regulating B-cell proliferation, differentiation, and immunoglobulin production including immunoglobulin E (IgE) (42, 43). Azithromycin is widely used in clinical practice; however, their mechanism of action is not fully understood. Given the involvement of the CD27 pathway in the pathophysiology of various T-lymphocyte-mediated inflammatory, autoimmune, and lymphoproliferative diseases, we examined the impact of AZM on CD27 regulation and potential consequences on CD4+ and CD8+ T-cell functions.

Herein, we provide mechanistic aspects of AZM-mediated T-lymphocyte function via downregulation of surface CD27 expression and their extracellular release as soluble CD27. In addition to defective CD27hi cell expansion, proliferation, and mTOR activity, AZM treatment resulted in depletion of type 1 immunity-associated effector CXCR3+CD27hi cells and diminished IFN-γ production by CD4+ and CD8+ T cells. These findings underscore future clinical implications of AZM in various T-lymphocyte-mediated diseases.

## 2 Methods

### 2.1 Peripheral blood mononuclear cell isolation and T-cell stimulation

Age-matched healthy donors (n = 8) without history of immune disorder or infection were recruited for this study. Peripheral blood mononuclear cells (PBMCs) were isolated—using Histopaque 1077 (Cat# 1077–100 ml, Sigma) density gradient centrifugation as described previously (20). Blood was overlaid on equal volume of Histopaque and centrifuged at 800g for 20 min at room temperature without break. Cells were washed twice with phosphate-buffered saline (PBS, Sigma-Aldrich). T-cell stimulation was performed with plate-bound anti-CD3 (4 µg/ml, Cat# 317302) and soluble anti-CD28 (2 µg/ml, Cat# 302902, BioLegend, USA). Briefly, PBMCs were seeded at a density of 1 × 10^6^ cells/ml in complete RPMI medium 1640 (Cat# 11875, Gibco) supplemented with 10% fetal bovine serum (FBS, Sigma-Aldrich), 100 U/ml penicillin, and 0.1 mg/ml streptomycin (Cat# 1514063, Gibco) in a 24-well culture plate for an indicated period. Prior written informed consents were obtained from each participant, and the study was conducted according to the Helsinki Declaration.

### 2.2 Monoclonal antibodies and immunophenotyping

For immunophenotyping, the following monoclonal antibodies were used: Alexa Fluor 700-anti-CD3 (clone OKT3, Cat# 65–0037-42, BioLegend, USA) or BUV737 anti-human CD3 (clone UCHT1, Cat# 612750, BD Biosciences), APC-eFluor 780-anti-CD4 (clone OKT4, Cat# 47–0048-42 eBiosciences), PE-Cy7-anti-CD4 (clone OKT4 Cat# 317414 BioLegend, USA) or BUV563 anti-CD4 (clone SK3, Cat# 612912, BD Biosciences), PE/Dazzle 594-anti-CD8 (clone SK1, Cat# 344744, eBiosciences, USA) or BV650 anti-CD8 (clone, RPA-T8, Cat# 563821, BD Biosciences), and PE-anti-CD27 (clone MT271, Cat# 555441, BD Biosciences, USA) or BUV395 anti-CD27 (Clone L128, Cat# 563815, BD Biosciences), APC-Cy7 anti-CD69 (clone FN50, Cat# 310914, BioLegend, USA), PE-Cy7-anti-CXCR3 (clone G025H7, Cat# 353720, BioLegend), or Alexa Fluor 488-anti-CXCR3 (clone G025H7 Cat# 353710, BioLegend). PBMCs were first stained with Live/Dead Zombie Violet fixable viability dye (Cat# L34955, BioLegend, USA) along with FcR Blocking Reagent-Human (Cat# 130–059-901, Miltenyi Biotec, Germany) for 15 min at 4°C in dark. Washed cells were stained with a panel of antibodies) for 25 min at 4°C in the dark. The cells were washed twice with FACS stain buffer (PBS+0.2% bovine serum albumen (BSA)+0.09% Azide, Cat# 554657, BD Pharmingen). The cells were acquired at BD FACSAria III or BD FACSSymphony A5 (BD Biosciences, USA) flow cytometer using BD FACSDiva software. Flow cytometric data were analyzed with Flow Jo software 9.5 (Tree Star). Single-stain compensation beads were used for multicolor compensation.

### 2.3 Cell proliferation assay

PBMCs were prelabeled with 5 µM carboxyfluorescein succinimidyl ester (CellTrace CFSE, Cell Proliferation Kit, Cat# C34554, Invitrogen, USA) in PBS for 8 min at room temperature in the dark with intermittent mixing. Cells were then washed twice with 10 ml of ice-cold complete RPMI 1640 medium containing 10% FBS. Briefly, cells were stimulated with plate-bound anti-CD3 and soluble anti-CD28 (eBiosciences) antibodies for 3 days in the presence or absence of azithromycin dihydrate (AZM, Cat# PZ0007, Sigma-Aldrich). A suboptimal dose of 40 µg/ml AZM was selected based on previous *in vitro* studies (19, 44, 45) and AZM titration analysis on T-cell viability (**Supplementary Figure S3**). Cells without anti-CD3/CD28 stimulation (unstim) were considered as negative control. On day 3, cells were harvested, washed with PBS, and stained with a panel of Alexa Fluor 700-anti-CD3 or BUV737-anti-human CD3, APC-eFluor 780-anti-CD4 or PE-Cy7-anti-CD4 or BUV563-anti-CD4, PE/Dazzle 594-anti-CD8 or BV650-anti-CD8, and PE-anti-CD27 or BUV395-anti-CD27 in FACS staining buffer for 25 minutes at 4°C. Washed cells were acquired using a flow cytometer.

### 2.4 Apoptosis assay

PBMCs were stimulated with anti-CD3/CD28 for 3 days in the presence or absence of AZM. Cells were stained with a panel of surface Alexa Fluor 700-anti-CD3 or BUV737-anti-human CD3, APC-eFluor 780-anti-CD4 or PE-Cy7-anti-CD4 or BUV563-anti-CD4, PE/Dazzle 594-anti-CD8 or BV650-anti-CD8, and PE-anti-CD27 or BUV395-anti-CD27 mAbs for 25 min at 4°C. Stained cells were washed twice with FACS stain buffer, and apoptosis assay was performed using FITC Annexin-V and 7-AAD Detection kit (Cat# 640922, BioLegend, USA) according to the manufacturer’s instructions.

### 2.5 Intracellular IFN-γ detection

PBMCs were stimulated with anti-CD3/CD28 for 3 days in the presence or absence of AZM. Brefeldin 10 µg/ml (Cat# 420601, BioLegend, USA) was added in the last 4 h of the culture. Cells were stained with a panel of surface Alexa Fluor 700-anti-CD3, PE-Cy7-anti-CD4, PE/Dazzle 594-anti-CD8, or and PE-anti-CD27 mAbs for 25 min at 4°C. Stained cells were washed and intracellularly stained with APC-eFluor 780-anti-IFN-γ mAb (clone 4SB3, Cat# 502529, BioLegend) using BD Cytofix/Cytoperm Fixation and Permeabilization solution kit (Cat# 554714, BD Biosciences, USA). To exclude possibility of unspecific IFN-γ staining, human Fc blocker or normal mouse serum was used during staining. Intracellular IFN-γ levels were detected with a flow cytometer.

### 2.6 Soluble CD27 quantification by ELISA

1 × 10^6^ PBMCs were stimulated with anti-CD3/CD28 for 3 days in the presence or absence of AZM. Culture supernatants were harvested and stored at −20°C until use. sCD27 protein levels were quantified using human CD27/TNFRS7 DuoSet ELISA kit (Cat# DY382–05, R&D Systems, USA) as per manufacturer’s instructions. All the samples were used in duplicates, and optical density was measured using a Spark microtiter plate reader (Tecan, USA) set at 450 nm.

### 2.7 Intracellular phospho-S6 ribosomal protein detection

To determine the mTOR activity, phosphorylated S6 ribosomal protein levels were detected as described previously (19). Briefly, PBMCs were stimulated with anti-CD3/CD28 for 22 h–24 h in the presence or absence of AZM. Cells were harvested, washed with PBS, and stained with Fixable Zombie violet Live/Dead stain along with an FcR blocker for 15 min. Cells were washed and surface stained with a panel of BUV737-anti-human CD3, BUV563-anti-CD4, BV650-anti-CD8, and BUV395-anti-CD27 mAbs as described above for 25 min at 4°C. Cells were fixed and permeabilized using eBiosciences FoxP3/Transcription factor staining kit (Cat# 00–5521-00, Thermo Fisher Scientific) and stained with anti-pS6RP-Alexa Fluor 488 (Ser235/236, clone 2F9, Cat# 4854, Cell Signaling Technologies). Stained cells were acquired by a flow cytometer.

### 2.8 Statistical analysis

For statistical analyses, we used GraphPad software 9.5.1. For comparing more than two groups, one-way analysis of variance (ANOVA) and Tukey’s multiple comparison test or two-way ANOVA followed by Šidàk multiple comparisons test were used. However, non-parametric Mann–Whitney U-test was used for two groups analysis. A p value of <0.05 was considered significant.

## 3 Results

### 3.1 AZM suppresses the expansion of CD27hi subset of TCR-activated T-lymphocytes

**Figure 1:**
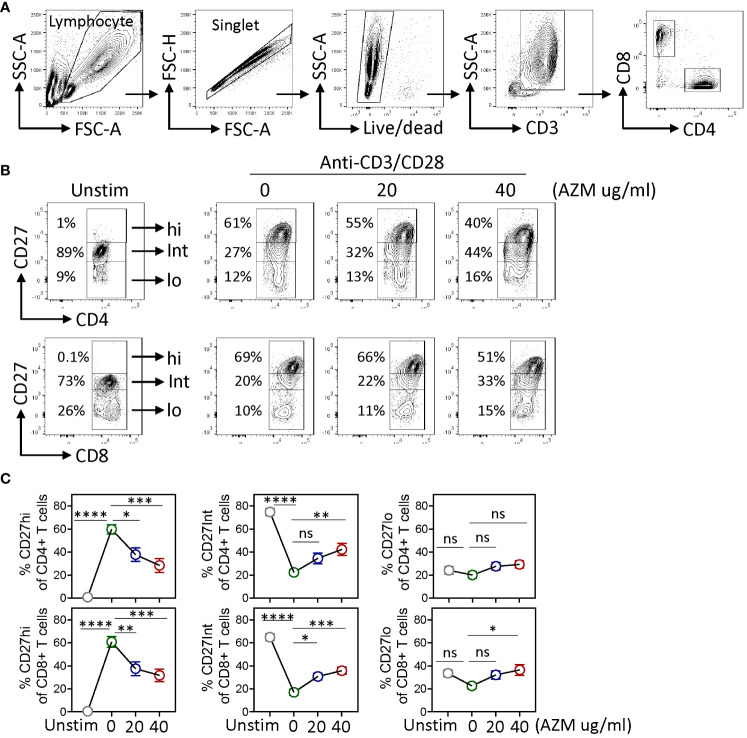
AZM suppresses CD27hi subset expansion. Anti-CD3/CD28-stimulated PBMCs were cultured for 3 days in the presence of 0 µg/ml, 20 µg/ml, and 40 µg/ml AZM. **(A)** Representative contour FACS plots showing the gating strategy; PBMCs were subsequently gated for singlet, live lymphocytes, CD3+, and CD4+ and CD8+ T-lymphocytes. **(B)** Representative contour FACS plots showing frequencies of CD27hi, CD27Int, and CD27lo subsets of CD4+ (upper panel) and CD8+ (lower panel) T cells. Numbers in each FACS plot denote the cell frequency in the respective CD27 gated subset. **(C)** Statistical plots showing percentage (mean ± SEM) of the CD27hi, CD27Int, and CD27lo subsets against indicated doses of AZM. Unstim stands for cells without anti-CD3/CD28 stimulation. Data presented are from n = 8 healthy individuals. Statistical significance was calculated using two-way ANOVA followed by Šidàk multiple comparisons test. *P < 0.05, **P < 0.01, ***P < 0.001, ****P < 0.0001, and ns stands for non-significant.

CD27 co-stimulation is known to play a pivotal role in T-lymphocyte expansion (22, 28). To investigate the impact of AZM on CD27 co-stimulation in context to T-lymphocyte expansion, healthy donors’ PBMCs were stimulated with anti-CD3/CD28 (TCR-activated) for 3 days in the presence or absence of AZM. Normally, in the absence of activation, the majority (70%–80%) of naive CD4+ and CD8+ T cells are positive for CD27 stain and that further increase following TCR activation. A stringent gating strategy was applied based on CD27 expression density on activated T cells with reference to resting (unstimulated), cells which normally lack CD27hi population. Our flow cytometry data revealed the appearance of three distinct subsets, CD27hi, CD27Int, and CD27lo on both CD4+ and CD8+ T cells (**Figures 1A, B**). Notably, in contrast to CD27Int and CD27lo subsets, CD27hi CD4+ and CD27hi CD8+ T cells showed significantly reduced frequencies following AZM treatment. AZM treatment resulted in a 2.5-fold decrease in CD27hi T cells compared with untreated cells (**Figures 1B, C**). Conversely, the reduction in the CD27hi subset was associated with elevated levels of CD27Int subset. However, we did not see any significant change in the CD27lo subset. These data suggest that AZM differentially affects the expansion of CD27 subsets and predominantly suppresses hyperactivated CD27hi CD4+ and CD27hi CD8+ T cells.

CD27 is considered as a reliable marker of memory T-lymphocyte differentiation (24, 25). To examine if AZM alters memory T-lymphocyte phenotype as well, we chose commonly defined CD45RA and CD27 markers to determine naïve (T_N_, CD45RA−CD27+), central memory (T_CM_, CD45RA+CD27+), effector memory (T_EM_, CD45RA−CD27−), and T-cell effector memory reactivated (T_EMRA_, CD45RA+CD27−). Flow analysis of CD4+ and CD8+ gated T cells did not show any significant impact of AZM on the above memory cell phenotype (**Supplementary Figure S1**).

### 3.2 AZM preferentially inhibits CD27hi subset proliferation by arresting cell division

**Figure 2:**
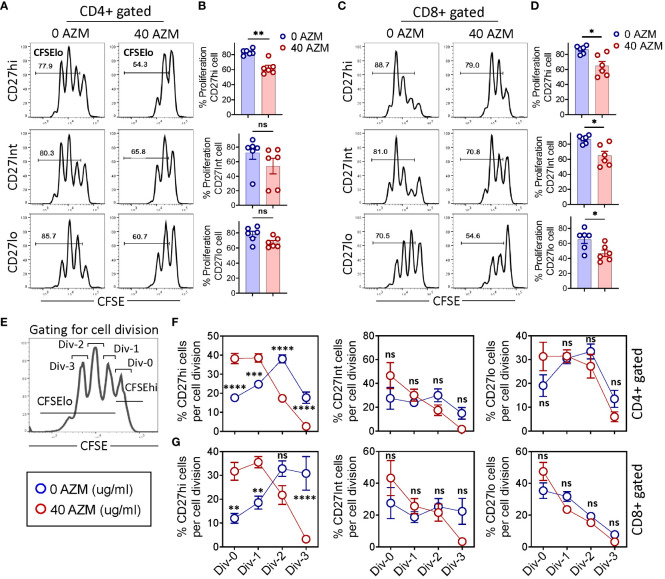
AZM preferentially arrests CD27hi subset cell division. CFSE-labeled PBMCs were stimulated with anti-CD3/CD28 in the presence or absence of 40 µg/ml AZM for 3 days. **(A)** Representative FACS histograms showing the levels of cell proliferation as determined by the CFSE dilution assay. Numbers in each histogram denote the percent proliferation (CFSElo cells) of CD27hi (top), CD27Int (middle), and CD27lo (bottom) subsets on CD4+ and **(C)** CD8+ T cells. **(B)** Scattered dot plots show percent (mean ± SEM) proliferation of respective CD27 subsets of CD4+ and **(D)** CD8+ T cells. **(E)** FACS histogram and schematic view of the gating strategy to determine the number of cell divisions. Div-0 (no division), Div-1 (one division), Div-2 (two divisions), and Div-3 (three divisions). **(F, G)** Statistical plots showing the proportion of proliferated cells per division (mean ± SEM) in CD27hi, CD27Int, and CD27lo subsets. Data presented are from n = 6 healthy individuals. Statistical significance was calculated using the Mann–Whitney (U) test for comparing two groups and two-way ANOVA followed by Šidàk multiple comparisons test for multiple groups. *P < 0.05, **P < 0.01, ***P < 0.001, ****P < 0.0001, and ns stands for non-significant.

To determine the mechanism of AZM-mediated defective CD27hi subset expansion, we measured the proliferative capacities across CD27 subsets by CFSE dilution. CFSE-labeled PBMCs were stimulated with anti-CD3/CD28 in the presence or absence of AZM and flow cytometry was performed on day 3 of culture. Flow cytometry analysis displayed a tendency of lower proliferation (CFSElo cells) by all the AZM-treated cells. This is evident from FACS histograms where TCR-activated cells had undergone at least three rounds of divisions (peak) on day 3 and that progressively reduced to either two or less than two with AZM (**Figures 2A, C**). However, it should be noted that among CD27 subsets of CD4+ T cell, only CD27hi cell and all the CD27 subsets of CD8+ T-cell subsets exhibited significant reduction in proliferation (**Figures 2B, D**).

To gain further insight on AZM-mediated impaired cell proliferation, we estimated the proportion of cells per cell division by gating histogram peaks (**Figure 2E**). AZM-treated CD27hi cells exhibited a drastic reduction in cell proportion at Div-2 and Div-3 of both CD4+ and CD8+ T cells (**Figures 2F, G**). Notably, this reduction was associated with marked elevation in the proportion of cells at Div-0 and Div-1. However, we did not observe any significant change with respect to CD27Int and CD27lo subsets of both CD4+ and CD8+ T-lymphocytes. The occurrence of higher proportion of CD27hi cells at Div-0 and Div-1 reflects the profound effect of AZM at the cell cycle level. These data suggest that AZM inhibits cell proliferation by blocking the entry of cells into the next round of cell division potentially by arresting cell cycle progression.

### 3.3 AZM induces apoptosis across CD27 subsets of activated T-lymphocytes

**Figure 3:**
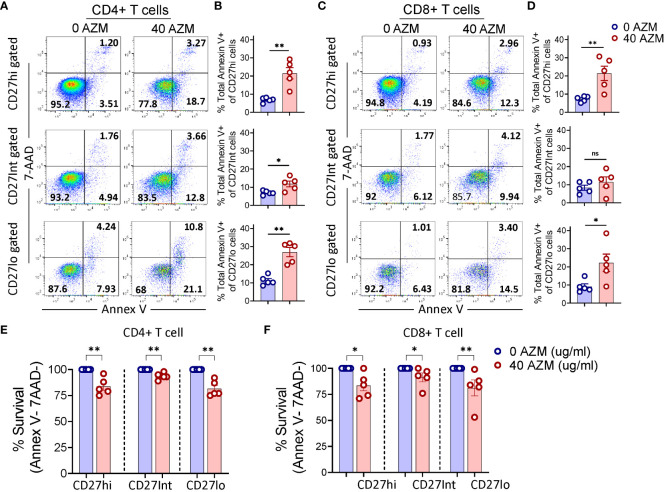
AZM promotes apoptosis of CD27 subsets. PBMCs were stimulated with anti-CD3/CD28 in presence or absence of 40 µg/ml AZM, and apoptosis was performed on day-3 using Annexin V and 7-AAD labelling. **(A)** Representative FACS plots showing percent apoptosis (Annexin V+ cells) on CD27hi, CD27Int, and CD27lo gated cells of CD4+ and **(C)** CD8+ T cells. **(B, D)** Scattered dot plots display total Annexin V+ cells (mean ± SEM) in respective CD27 subsets. **(E, F)** Scattered dot plots show % survival (Annex V-7AAD- cells) of CD27hi, CD27Int, and CD27lo subsets of CD4+ and CD8+ T cells. Percent cell survival was calculated by considering AZM untreated cells survival as hundred percent. Data presented are from n = 5 healthy individuals. Statistical significance was calculated using Mann–Whitney (U) test. *P < 0.05, **P < 0.01, and ns stands for non-significant.

One of the potential mechanisms of AZM-mediated CD4+ T-cell suppression is through apoptotic induction (10, 16). Given the contribution of CD27 signaling in T-lymphocyte survival (46), we tested if apoptosis contributes to proliferative impairment of CD27hi subsets. TCR-stimulated PBMCs were subjected to apoptosis assay using viability dye Annexin V and 7-AAD. Our flow cytometry data showed an increased level of total Annexin V+ (apoptotic) cells across the CD27 subsets following AZM exposure (**Figures 3A, C**). This phenotype was reflected in most of the statistical plots of CD27 subsets of both CD4+ and CD8+ T cells (**Figures 3B, D**). Moreover, similar patterns were also observed when we analyzed the cell survival (annex V-7-AAD- cells) of these subsets (**Figures 3E, F**). These results suggest that all the TCR-stimulated CD27 subsets except CD8+ CD27Int show sensitivity toward AZM-mediated apoptosis.

### 3.4 AZM downregulates CD27 expression on TCR-activated T-lymphocytes

**Figure 4:**
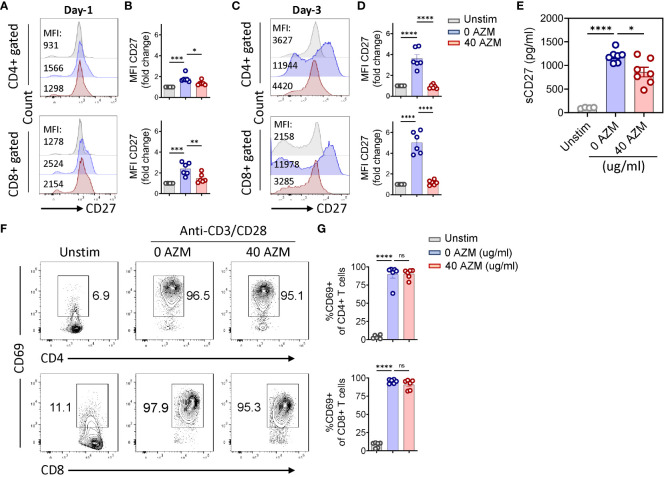
AZM downregulates CD27 expression on activated T-lymphocytes. Cells were stimulated with anti-CD3/CD28 for the indicated period in the presence or absence of 40 µg/ml AZM. Representative FACS histogram showing mean fluorescence intensities (MFIs) of CD27 on CD4+ and CD8+ gated T cells on day 1 **(A)** and day 3 **(C)**. Scattered dot plots show fold change in the MFI (mean ± SEM) normalized to unstimulated cells of CD4+ and CD8+ T cells on day 1 **(B)** and day 3 **(D)**. Soluble CD27 concentration was measured in the culture supernatant using ELISA. **(E)** Scattered dot plot showing the concentration of sCD27 pg/ml (mean ± SEM). Data represent unstim (n = 4), 0 AZM and 40 AZM (n = 7 individuals each). **(F)** Representative contour FACS plot showing activation status, CD69 expression levels on CD4+ (upper panel), and CD8+ T cells (lower panel). Unstim cells serve as negative control. **(G)** Scattered dot plot showing percent CD69+ (mean ± SEM) cells on CD4+ and CD8+ T cells. Data presented are from n = 6 **(B, D, G)** and n = 7 **(E)** healthy individuals. Statistical significance was calculated using one-way ANOVA followed by Tukey’s multiple comparison test. *P < 0.05, **P < 0.01,***P < 0.001, ****P < 0.0001, and ns stands for non-significant.

CD27 costimulation plays a crucial role in T-lymphocyte proliferation and survival (28, 29). Thus, we sought to determine if AZM regulates CD27 expression to modulate T-lymphocyte expansion or proliferation. Our flow cytometry analysis (**Figures 4A, C**) of AZM-treated T-lymphocytes clearly showed significantly lower levels of surface CD27 expression overtime (day 1 and day 3) compared with resting or unstimulated cells of CD4+ and CD8+ gated cells. In addition to downregulation of CD27 expression on day 1, AZM exposure resulted in twofold lesser CD27 expression on day 3 as determined by mean fluorescence intensity (MFI) on both CD4+ and CD8+ gated T cells (**Figures 4B, D**). These data suggest that AZM can downregulate surface CD27 expression on TCR-activated T-lymphocyte to potentially inhibit the cell proliferation eventually the expansion.

CD27 is also released as soluble CD27 in the culture medium following TCR activation of T lymphocytes (37). To test this phenomenon, we measured the levels of sCD27 in the culture supernatant. Our ELISA results clearly showed significantly low levels of sCD27 following AZM treatment (**Figure 4E**) indication. AZM not only inhibits the surface CD27 but also decreases its release. Next, we examined if AZM affects the activation status of T lymphocytes by measuring the levels of early lymphocyte marker CD69 on day 1. Contour FACS plots of both AZM-treated and untreated CD4+ and CD8+ T cells showed similar activation status (**Figures 4F, G**) as statistic plots also showed no significant change in activation. That means AZM downregulates CD27 expression without affecting the activation status of the cells.

### 3.5 AZM significantly inhibits the mTOR activity of the CD27hi subset

**Figure 5:**
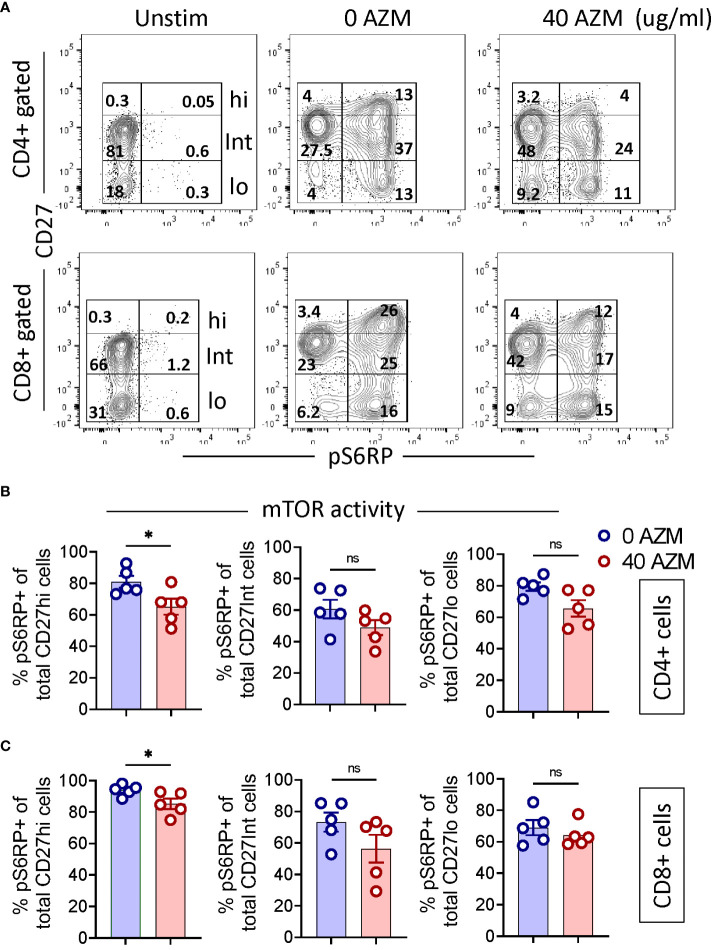
AZM attenuates mTOR activity of CD27hi subset. Cells were stimulated with anti-CD3/CD28 in the presence or absence of 40 µg/ml AZM for 24 h. mTOR activity was determined by measuring the levels of phosphorylated S6 ribosomal protein (pS6RP). Resting cells without anti-CD3/CD28 stimulation (Unstim) are taken as negative control and to set CD27hi subset gating. **(A)** Representative contour FACS plots show levels (%) of pS6RP in CD27hi, CD27Int, and CD27lo subsets of CD4+ (upper panel) and CD8+ T cell (lower panel). **(B)** Scattered dot plots displaying % pS6RP+ cells (mean ± SEM) in total CD27hi, CD27Int, and CD27lo subset of CD4+ and **(C)** CD8+ T cells. Data presented are from n = 5 healthy individuals. Statistical significance was calculated using the Mann–Whitney (U) test. *P < 0.05, and ns stands for non-significant.

Previous studies have shown inhibition of mTOR activity as one of the mechanisms of AZM-mediated defective T-lymphocyte proliferation (17, 18). However, its effects on various CD27 subsets of activated T-lymphocyte are elusive. We sought to determine if AZM differentially affects the mTOR activities of TCR-activated CD27 subsets. To assess this, intracellular levels of phosphorylated S6 ribosomal protein (pS6RP) were measured using flow cytometry. FACSplots displayed elevated levels of pS6RP following TCR activation and that subsequently attenuated following AZM treatment (**Figure 5A**). Although there is a general tendency of diminished pS6RP levels across CD27 subsets, only CD27hi subsets of both CD4+ and CD8+ T-lymphocyte could reach a significant value (p < 0.05) (**Figures 5B, C**). These data suggest that AZM suppresses CD27hi cell proliferation potentially by inhibiting S6RP phosphorylation or mTOR activity.

### 3.6 AZM treatment results in depletion of the type-1 effector CXCR3+ CD27hi subset

**Figure 6:**
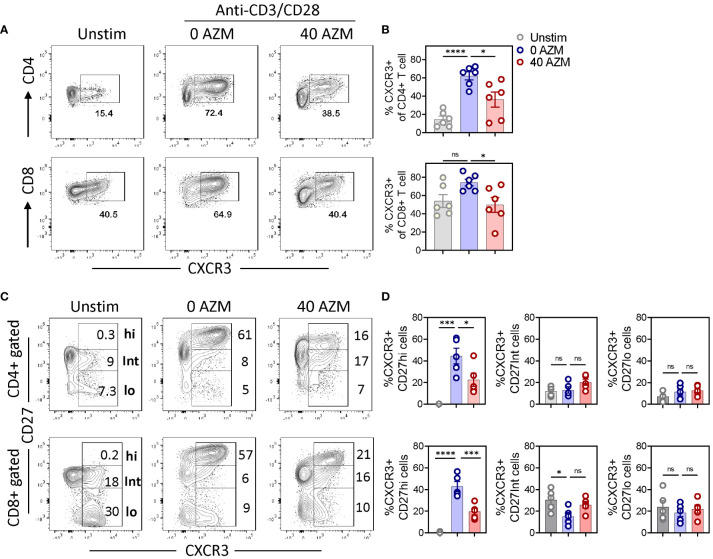
AZM triggers depletion of the CXCR3+ CD27hi subset. PBMCs were stimulated for 3 days in the presence or absence of 40 µg/ml AZM. **(A)** Representative contour FACS plots showing percentage of CXCR3+ cells on bulk CD4+ (upper panel) and CD8+ T cells (lower panel). **(B)** Scattered statistical plots showing percent CXCR3+ cells (mean ± SEM) on respective CD4+ and CD8+ gated T cells. **(C)** Representative contour FACS plots showing levels of CXCR3+ cells on CD27hi, CD27Int, and CD27lo subsets of CD4+ (upper panel) and CD8+ T cells (lower panel). Unstimulated (Unstim) cells served as control and to set CD27hi gating. **(D)** Scattered dot plots showing percent CXCR3+ cells (mean ± SEM) on CD27hi, CD27Int, and CD27lo subset of respective CD4+ and CD8+ gated T cells. Data presented are from n = 6 healthy individuals. Statistical significance was calculated using one-way ANOVA followed by Tukey’s multiple comparison test. *P < 0.05, ***P < 0.001, ****P < 0.0001, and ns stands for non-significant.

CD27 costimulation promotes differentiation of CD4+ T cell into Th1 effectors (33) and CD8+ T cell into CTLs (34) by inducing type-1 transcription factor T-bet to transcribe IFN-γ and chemokine receptor CXCR3 (35, 36, 47). To assess this, we investigated the effect of AZM on levels of type-1 effector signature chemokine receptor CXCR3 and cytokine IFN-γ on various CD27 subsets. Flow cytometry data clearly exhibited significant reduction in the frequency of CXCR3+ CD4+ and CXCR3+CD8+ T cells (**Figures 6A, B**). Notably, in contrast to CD27Int and CD27lo subsets, we observed a marked reduction in the frequency of the effector CXCR3+CD27hi subset of both CD4+ and CD8+ T cells (**Figures 6C, D**). These data suggest that AZM exerts the immunosuppressive activity of both Th1 and CTLs by selective targeting of inflammatory or effector CXCR3+CD27hi subset that potentially infiltrates to the site of inflammation to orchestrate immune reactions.

### 3.7 AZM diminishes type-1 effector cytokine IFN-γ production by CD27 subsets

T-lymphocytes, in particular Th1 and CTLs, are known to produce large amounts of effector cytokine IFN-γ following activation. We and others have previously shown inhibition of IFN-γ production by CD4+ T cells following AZM treatment (18, 20). We tested this effect on IFN-γ-producing capacities of various CD27 subsets by employing the intracellular staining and flow cytometry approach. Significantly reduced IFN-γ production was observed across the CD27 subsets of both CD4+ and CD8+ T cells following AZM treatment (**Supplementary Figure S2**). However, resting (unstimulated) cells do not express IFN-γ (data not shown). These data suggest that AZM globally inhibits the intracellular IFN-γ production by T-lymphocytes.

## 4 Discussion

Previous studies including ours have shown the immunomodulatory behavior of AZM on TCR-activated T-lymphocytes (18, 20, 48). However, its effects on T-lymphocyte costimulatory molecules are unknown. Very recently, we reported downregulation of ICOS and OX40 expression as one of the potential mechanisms for defective T-lymphocyte proliferation (19). Given the pivotal role of CD27 costimulation in T-lymphocyte expansion, survival, and effector function, we investigated the immunomodulatory effects of AZM on CD27 and potential consequence on CD4+ and CD8+ cell function. Based on the levels of surface CD27 expression on TCR-activated T-lymphocytes, our flow cytometry data revealed appearance of three distinct subpopulations referred to as CD27hi, CD27Int, and CD27lo subsets. It seems that a proportion of CD27Int (resting cells) population expanded and acquired a higher CD27 expression to become the CD27hi subset as our flow cytometry data clearly showed marked reduction in the frequency of resting CD27Int cells without affecting CD27lo subsets. This inverse correlation between CD27hi and CD27Int cell numbers points toward a differential behavior of AZM on these subsets. This phenomenon perhaps could be a mechanism of AZM to bring down the elevated levels of CD27hi cells to compensate the loss in the CD27Int subset. However, further investigation is required to confirm this effect.

The defective CD27hi expansion was related to impaired cell proliferation as AZM-treated cells displayed lesser CFSElo cells. TCR-activated CD27 subsets of both CD4+ and CD8+ T cells underwent at least three rounds of cell division on day 3 which reduced to ≤2 divisions with AZM. However, a more pronounced effect of AZM was seen at the cell division level where AZM exposure preferentially inhibited the cell division progression of the CD27hi subset either by retaining the cells in undivided state (Div 0) or by blocking the entry of cells into subsequent divisions. These data suggest that AZM inhibits the cell proliferation of the CD27hi subset by arresting cell cycle progression, which is in agreement with a previously reported study on rapamycin-mediated cell cycle arrest of T cells involving mTOR (49).

AZM is known to induce T-lymphocyte apoptosis (18, 20). In agreement with this, AZM treated CD27 subsets of both CD4+ and CD8+ cells exhibited enhanced apoptosis as determined by elevated levels of total annexin V+ cells. This pattern was also reflected in cell survival analysis of CD27 subsets as well. It appears that elevated apoptosis may not be the major factor contributing to defective CD27hi cell expansion.

Our result describes defective CD27hi cell expansion or proliferation with AZM. Given the regulatory role of CD27 costimulation in T-lymphocyte proliferation, survival, and effector function (25, 27–29, 34, 50), we assessed the surface expression levels of CD27 on a per cell basis. FACS histograms clearly showed significant downregulation of surface CD27 expression on AZM-treated CD4+ and CD8+ T cells. Apart from downregulating surface CD27 on activated T-lymphocytes, AZM also inhibited the release of the soluble form of CD27 in culture supernatant of AZM-treated cells. A finding underscored the future clinical use of AZM as adjunct therapy in chronic inflammatory diseases such as RA and SLE where elevated levels of sCD27 are associated with disease pathogenesis (38, 39). However, the downregulation of CD27 expression was independent of the activation status of T-lymphocytes as early activation marker CD69 did not show any significant change with AZM. Thus, lower CD27 surface density on AZM-treated cells may contribute to altered CD27 signaling leading to CD27hi cell dysfunction.

AZM-mediated mTOR inhibition is considered as one of the mechanisms for T-lymphocyte dysfunction via the PI3K/Akt/mTOR pathway (51). Notably, CD27hi subsets from both TCR-activated CD4+ and CD8+ T cells showed significantly lower S6RP phosphorylation or mTOR activity than other subsets, thus supporting the notion that lower mTOR activity in the CD27hi subset could be one of the factors contributing to defective cell proliferation (49). In other words, AZM seems to preferentially target cells that are metabolically active, proliferating, and exhibit higher mTOR activity, as observed in the case of CD27hi cells.

Multiple studies have shown implications of CXCR3+ T-lymphocytes in inflammation (38, 39) and acute graft reject (52). Given the crucial role of CD27 signaling in regulating CXCR3 expression via type-1 transcription factor T-bet (35, 47), and differentiation of IFN-γ-secreting Th1 and Tc1 cells, the selective loss of the effector CXCR3+CD27hi subset following AZM treatment could be one of the potential anti-inflammatory mechanisms to limit the activation and trafficking of CXCR3+ T-cell effectors to inflammation loci. Some of the limitations of this study include non-testing of AZM on sorted CD27 subsets to have cell-intrinsic effects, which is basically our next line of investigation. Although we observed AZM to efficiently suppress CXCR3 expression on effector T cells, their association with type-1 signature transcription factor T-bet needs to be examined, including ex vivo analysis of the T-cell phenotype and function on peripheral blood of AZM-treated patients.

From a clinical perspective, these findings shed light on potential mechanisms operating in patients on prophylactic AZM treatment for various inflammatory airway diseases including COPD, asthma, and idiopathic pulmonary fibrosis (IPF) (1–3). Given the role of CD27–CD70 interaction in B-cell activation and immunoglobulin E (IgE) production (43), observed AZM-mediated CD27 downregulation may provide a new strategy against allergic diseases. Very recently, a clinical trial study has reported AZM-mediated increased relapse of malignancies after allogeneic hematopoietic stem cell transplantation (53). In this regard, our findings on AZM-mediated downregulation of CD27, inhibition of metabolic sensor mTOR, and loss of effector CXCR3+CD27hi Tc1 could perhaps provide possible explanations to this relapse.

## 5 Conclusions

This study demonstrates a new mechanism of AZM-mediated T-lymphocyte modulation by targeting the CD27 pathway. These findings could perhaps explain one of the potential clinical benefits of prophylactic AZM regimens in various airway diseases. Given the contribution of the CD27 pathway in Th1- and Tc1-mediated pathogenesis of various inflammatory and autoimmune diseases, and graft rejection, AZM may serve as adjunct therapy against these anomalies. Further investigation is warranted to translate these findings into clinical settings.

## Data Availability

The original contributions presented in the study are included in the article/**Supplementary Material**. Further inquiries can be directed to the corresponding authors.

## Supplementary material

Supplementary Figure 1 AZM does not alter T-lymphocyte memory phenotype. **(A)** Representative FACS plots showing effects of AZM on memory T-lymphocytes on day-3. Major memory T -lymphocyte subsets, central memory (T_CM_, CD27+CD45RA-), naïve (T_N_, CD27+CD45RA+), T cell effector memory cell re-expressing CD45RA (T_EMRA_, CD27-CD45RA+) and effector memory (T_EM_, CD27-CD45RA-) on CD4+ and CD8+ gated T cells. Numbers in each FACS plot quadrant denote the frequency (%) of memory subset. **(B)** Scattered dot plots displaying mean ± SEM memory cell frequency. Data presented are from n=7 healthy individuals. Statistical significance was calculated using Mann-Whitney (U) test. ns stands for non-significant.

Supplementary Figure 2 AZM diminishes type-1 effector cytokine IFN-γ production by CD27 subsets. Representative FACS plots showing intracellular IFN-γ production in CD27hi (upper), CD27Int (middle) and (lower) panels of CD4+ **(A)** and CD8+ **(C)** gated T cells. **(B, D)** Scattered dot plots display % IFN-γ (mean ± SEM) production by corresponding CD27 subsets of CD4+ and CD8+ gated T cells. Data presented are from n=5 healthy individuals. Statistical significance was calculated using Mann-Whitney (U) test. **P<0.01 and ns stands for non-significant.

Supplementary Figure 3 AZM dose response on T cell viability. PBMCs were stimulated for 3-days in presence of 0, 20, 40 or 80 ug/ml AZM. **(A)** Representative contour FACS plots showing percentage of live cells, based on FSC-A and 7-AAD. **(B)** Representative bar diagram shows the percent T cell survival, calculated by considering AZM untreated (0 AZM) cells survival as hundred percent. Data represented is from one of the two healthy individuals analyzed.
