# Supplementary figures and images for: Azithromycin targets the CD27 pathway to modulate CD27hi T-lymphocyte expansion and type-1 effector phenotype
Source: Front Immunol. 2024 Aug 15;15:1447625. doi: 10.3389/fimmu.2024.1447625 (PMC11357905; doi:10.3389/fimmu.2024.1447625)

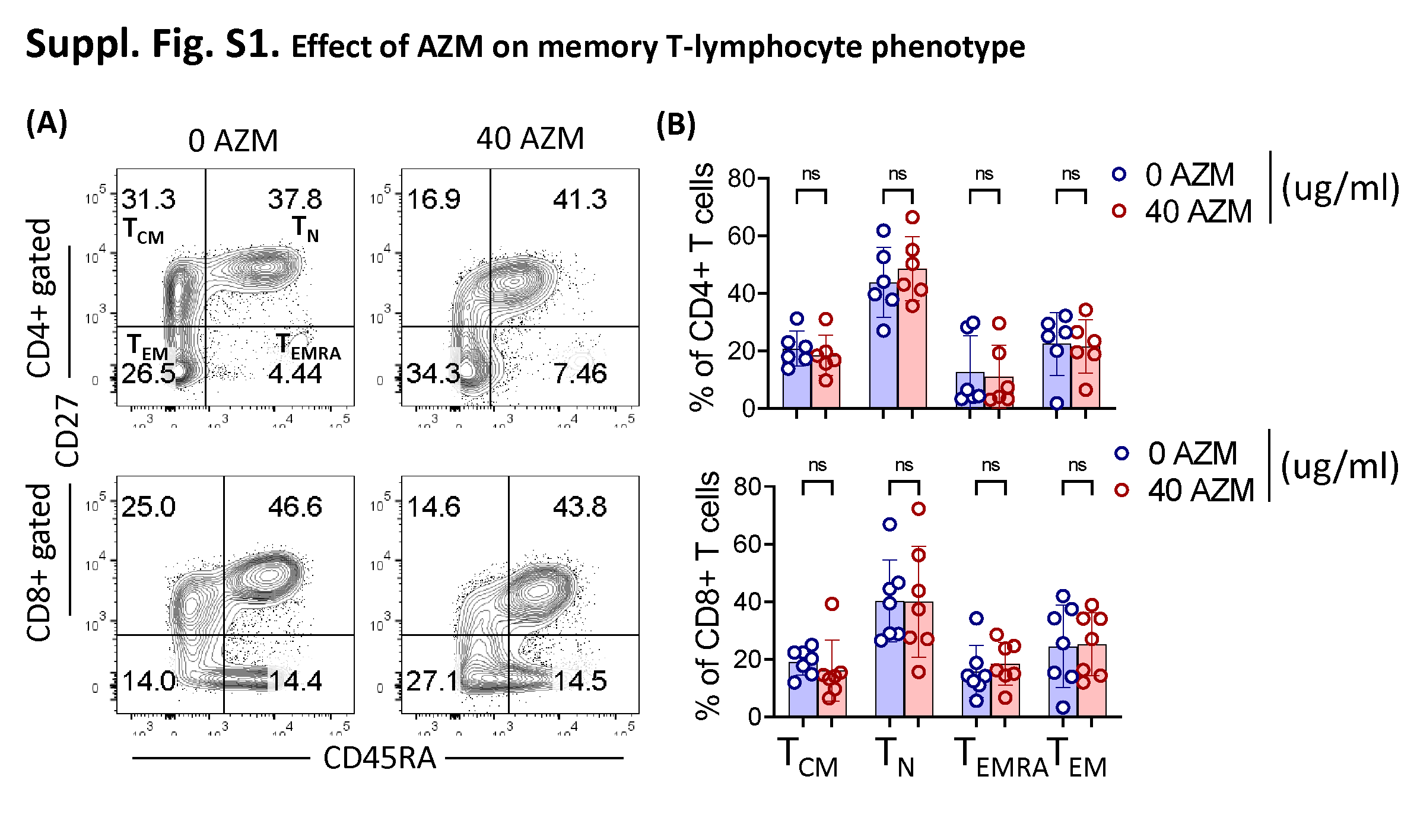

Supplement: Supplementary Figure 1 — AZM does not alter T-lymphocyte memory phenotype. (A) Representative FACS plots showing effects of AZM on memory T-lymphocytes on day-3. Major memory T -lymphocyte subsets, central memory (TCM, CD27+CD45RA-), naïve (TN, CD27+CD45RA+), T cell effector memory cell re-expressing CD45RA (TEMRA, CD27-CD45RA+) and effector memory (TEM, CD27-CD45RA-) on CD4+ and CD8+ gated T cells. Numbers in each FACS plot quadrant denote the frequency (%) of memory subset. (B) Scattered dot plots displaying mean ± SEM memory cell frequency. Data presented are from n=7 healthy individuals. Statistical significance was calculated using Mann-Whitney (U) test. ns stands for non-significant. [file Image1.tif]

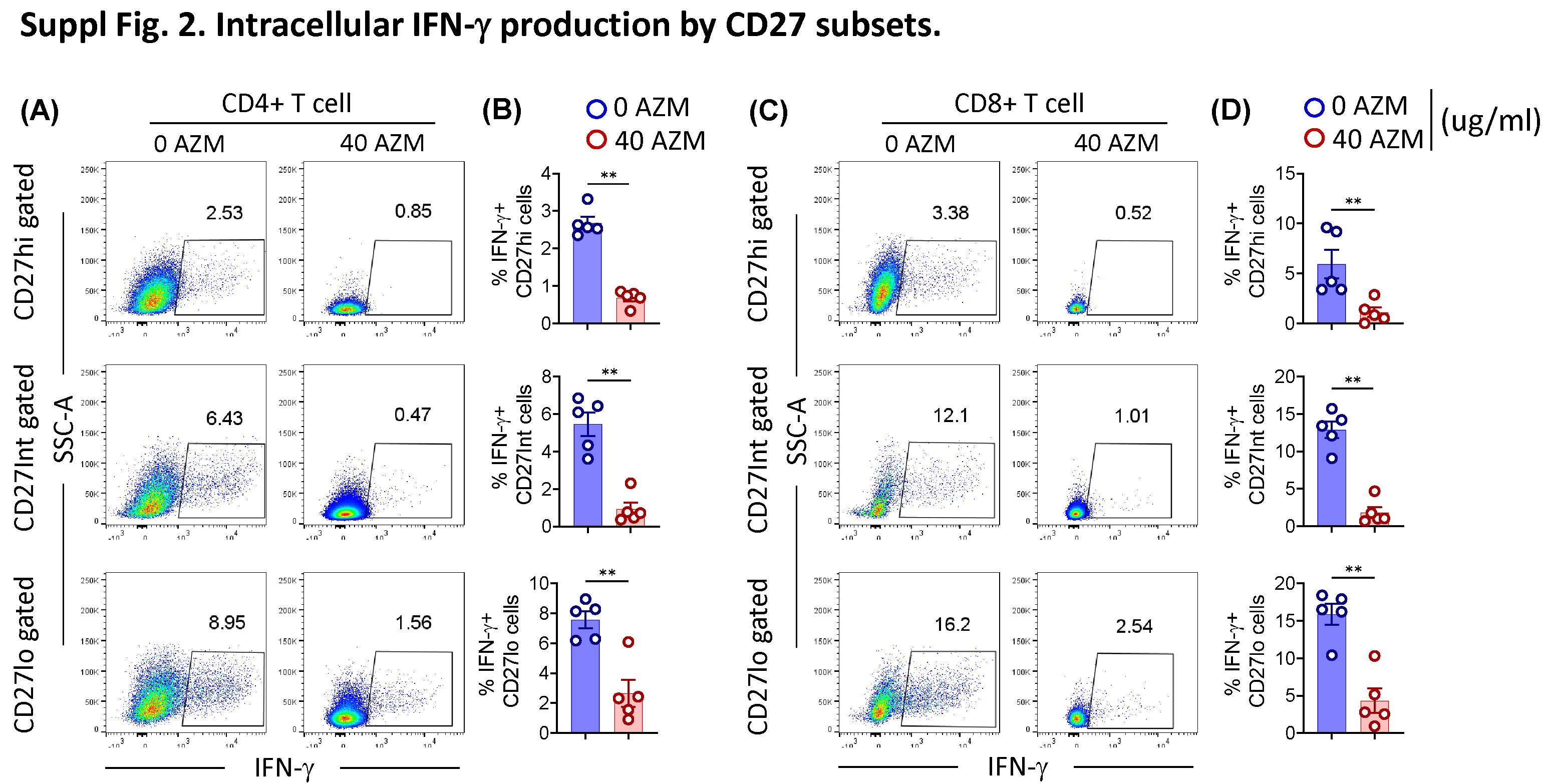

Supplement: Supplementary Figure 2 — AZM diminishes type-1 effector cytokine IFN-γ production by CD27 subsets. Representative FACS plots showing intracellular IFN-γ production in CD27hi (upper), CD27Int (middle) and (lower) panels of CD4+ (A) and CD8+ (C) gated T cells. (B, D) Scattered dot plots display % IFN-γ (mean ± SEM) production by corresponding CD27 subsets of CD4+ and CD8+ gated T cells. Data presented are from n=5 healthy individuals. Statistical significance was calculated using Mann-Whitney (U) test. **P<0.01 and ns stands for non-significant. [file Image2.tif]

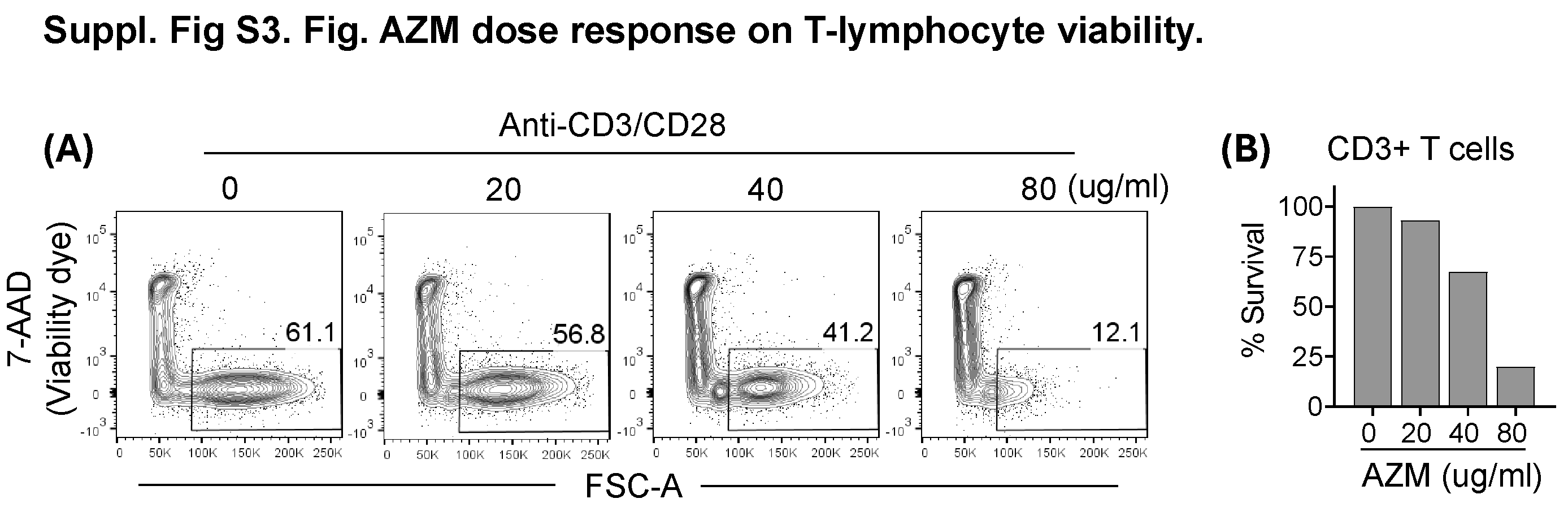

Supplement: Supplementary Figure 3 — AZM dose response on T cell viability. PBMCs were stimulated for 3-days in presence of 0, 20, 40 or 80 ug/ml AZM. (A) Representative contour FACS plots showing percentage of live cells, based on FSC-A and 7-AAD. (B) Representative bar diagram shows the percent T cell survival, calculated by considering AZM untreated (0 AZM) cells survival as hundred percent. Data represented is from one of the two healthy individuals analyzed. [file Image3.tif]
